# Molecular Characterization of Somatic Alterations in Dukes’ B and C Colorectal Cancers by Targeted Sequencing

**DOI:** 10.3389/fphar.2017.00465

**Published:** 2017-07-18

**Authors:** Shafina-Nadiawati Abdul, Nurul-Syakima Ab Mutalib, Khor S. Sean, Saiful E. Syafruddin, Muhiddin Ishak, Ismail Sagap, Luqman Mazlan, Isa M. Rose, Nadiah Abu, Norfilza M. Mokhtar, Rahman Jamal

**Affiliations:** ^1^UKM Medical Molecular Biology Institute Universiti Kebangsaan Malaysia, Kuala Lumpur, Malaysia; ^2^Thermo Fisher Scientific Shah Alam, Malaysia; ^3^Department of Surgery, Faculty of Medicine, Universiti Kebangsaan Malaysia Kuala Lumpur, Malaysia; ^4^Department of Pathology, Faculty of Medicine, Universiti Kebangsaan Malaysia Kuala Lumpur, Malaysia; ^5^Department of Physiology, Faculty of Medicine, Universiti Kebangsaan Malaysia Kuala Lumpur, Malaysia

**Keywords:** colorectal cancer, next generation sequencing, Ion Ampliseq Comprehensive Cancer Panel, *APC*, *SYNE1*, precision medicine

## Abstract

Despite global progress in research, improved screening and refined treatment strategies, colorectal cancer (CRC) remains as the third most common malignancy. As each type of cancer is different and exhibits unique alteration patterns, identifying and characterizing gene alterations in CRC that may serve as biomarkers might help to improve diagnosis, prognosis and predict potential response to therapy. With the emergence of next generation sequencing technologies (NGS), it is now possible to extensively and rapidly identify the gene profile of individual tumors. In this study, we aimed to identify actionable somatic alterations in Dukes’ B and C in CRC via NGS. Targeted sequencing of 409 cancer-related genes using the Ion Ampliseq^TM^ Comprehensive Cancer Panel was performed on genomic DNA obtained from paired fresh frozen tissues, cancer and normal, of Dukes’ B (*n* = 10) and Dukes’ C (*n* = 9) CRC. The sequencing results were analyzed using Torrent Suite, annotated using ANNOVAR and validated using Sanger sequencing. A total of 141 somatic non-synonymous sequence variations were identified in 86 genes. Among these, 64 variants (45%) were predicted to be deleterious, 38 variants (27%) possibly deleterious while the other 39 variants (28%) have low or neutral protein impact. Seventeen genes have alterations with frequencies of ≥10% in the patient cohort and with 14 overlapped genes in both Dukes’ B and C. The adenomatous polyposis coli gene (APC) was the most frequently altered gene in both groups (*n* = 6 in Dukes’ B and C). In addition, *TP53* was more frequently altered in Dukes’ C (*n* = 7) compared to Dukes’ B (*n* = 4). Ten variants in *APC*, namely p.R283^∗^, p.N778fs, p.R805^∗^, p.Y935fs, p.E941fs, p.E1057^∗^, p.I1401fs, p.Q1378^∗^, p.E1379^∗^, and p.A1485fs were predicted to be driver variants. APC remains as the most frequently altered gene in the intermediate stages of CRC. Wnt signaling pathway is the major affected pathway followed by P53, RAS, TGF-β, and PI3K signaling. We reported the alteration profiles in each of the patient which has the potential to affect the clinical decision. We believe that this study will add further to the understanding of CRC molecular landscape.

## Introduction

Colorectal cancer (CRC) is the third most common cause of cancer-related deaths worldwide (Siegel et al., 2016). Its incidence has been increasing rapidly in some areas of the world, including Asia (Jemal et al., 2011). In Malaysia, CRC has become the second most common cancer in both men and women where men were slightly more affected than women [1.1:1] (Ferlay et al., 2012). According to Lim (2014), the cumulative lifetime risk of developing CRC for Malaysians was 1:38 in men and 1:50 in women. The Malaysian Chinese has twice higher risk compared to Indian and Malay (Lim, 2014).

Survival rate of CRC is highly dependent on the stage of disease at diagnosis (Jemal et al., 2011). The 5-year survival rate for early stage is 60–95% but this dramatically decreases to 35% for those with lymph nodes involvement, indicating that early screening and treatment are crucial to improve management of CRC patients (Sengupta et al., 2008). Dukes’ staging (Dukes, 1932) and TNM classification (Version 7) (Edge et al., 2009) are the two staging systems used in CRC worldwide. Until today, CRC staging is based solely on simple clinicopathological features such as bowel wall penetration and lymph node metastasis. Current standard therapies for CRC patients depend on the stages. For instance, radical surgery followed by adjuvant chemotherapy is the standard practice for Dukes’ C patients (Hagan et al., 2013). However, the treatment remains controversial for Dukes’ B patients because the toxicities may outweigh its benefits (Benson et al., 2004). Nevertheless, due to heterogeneity nature of cancers, clinical outcome of patients with the same histo-clinical staging could also differ.

Generally, adjuvant treatment for CRC consists of 5 fluorouracil (5-FU) and Oxaliplatin (Piedbois et al., 1992; de Gramont et al., 2000; Saltz et al., 2000; Fuchs et al., 2008). A modern choice of the treatment has emerged by using targeted personalized therapy where the molecular profiling of each individual tumor is needed (Gray et al., 2011). Due to the heterogenous nature of CRCs, identification of biomarkers for targeted therapies is now highly recommended as the genetic and epigenetic alterations have been demonstrated to correlate with prognosis and treatment responses (Silvestri et al., 2013). For instance, CRC tumors with KRAS alterations in codons 12 and 13 are irresponsive to regularly used drugs targeting EGFR, and tumors with chromosome instability frequently found in majority of CRCs are often associated with multi-drug resistance (Lee et al., 2011).

Identification of somatic alterations is one of the key approaches to understand the molecular mechanisms of CRC and for the development of novel therapeutics. Deep sequencing via NGS technology is revolutionizing our understanding of somatic changes occurring in the cancer genome. In this study, we utilized the 409-gene targeted sequencing assay, the Ampliseq Comprehensive Cancer Panel v2 (CCPv2), performed on the Ion Torrent PGM, to identify actionable somatic alterations in non-metastatic CRC.

## Materials and Methods

### Clinical Specimens, Nucleic Acids Extraction and Quality Assessments

This study was approved by the Universiti Kebangsaan Malaysia Research Ethics Committee (Reference number: UKM 1.5.3.5/244/UMBI-004-2012). A total of 38 fresh frozen CRC tissues from Duke’s B and Duke’s C and the corresponding adjacent normal epithelial cells were collected during surgery from patients admitted to the Universiti Kebangsaan Malaysia Medical Centre (UKMMC), Kuala Lumpur, Malaysia. Confirmation of diagnosis and metastasis status were made based on histopathological report. All subjects gave written informed consent. The tissues were kept frozen in liquid nitrogen until subjected to cryosectioning. Hematoxylin and Eosin (H&E) staining was performed and slides were reviewed by the pathologist. Only cancer tissues that contained more than 80% tumor cells with less than 20% necrosis were included in this study. The normal specimens were confirmed to be free from tumor or inflammatory cells.

DNA was extracted from the tissues using the QIAamp^®^ DNA Mini Kit (Qiagen, Valencia, CA, United States). Nucleic acid quality and quantity were assessed using the Qubit Fluorometer (Invitrogen, Carlsbad, CA, United States), NanoDrop 2000 Spectrophotometer (NanoDrop Technologies, Wilmington, DE, United States) and agarose gel electrophoresis. The highly intact and non-degraded RNA-free genomic DNA was subjected to library preparation prior to sequencing. Total RNA was extracted from the cell lines with plasmid constructs using RNeasy Mini Kit (Qiagen, Valencia, CA, United States) according to the manufacturer’s protocol. The RNA quantity and purity were determined using the NanoDrop 20000 spectrophotometer (NanoDrop Technologies, Wilmington, DE, United States) and RNA with OD260/280 ratios of 1.8–2.1.

### Cell lines and Culture Conditions

The SW480 and 293T cells were purchased from the American Type Culture Collection (Manassas, VA, United States). SW480 cells were maintained in RPMI (Gibco, Life Technologies) supplemented with 10% fetal bovine serum (FBS) (Gibco, Life Technologies) while 293T cells were maintained in DMEM, with 10% FBS. Both cell lines were maintained at 37°C in a humidified incubator containing 5% CO_2_.

### Microsatellite Instability

The microsatellite status of each tumor was determined via immunohistochemical staining on the formalin fixed paraffin embedded (FFPE) tissue sections using the protocol from EnVision^TM^ FLEX Mini Kit, High pH (Code No. K8023, Dako, Denmark).

### Library Preparation, Emulsion PCR and Ion Torrent PGM^TM^ Sequencing

The Ion Ampliseq^TM^ Comprehensive Cancer Panel V2 (Life Technologies, Guilford, CT, United States) which covers 409 oncogenes and tumor suppressor genes relevant for cancer was used for library preparation. Briefly, DNA amplification was carried out with 10 ng DNA from each sample using the Ion Ampliseq HiFi Master Mix. Sequencing adaptors that enable sample multiplexing were ligated to the amplicon using the Ion adapter. The adapter-ligated amplicons (library) were purified using the Agencourt^®^ AMPure^®^ XP beads (BD Bioscience, United States). The library was subjected to the second round of amplification using the Platinum PCR Super Mix High Fidelity and Library Amplification Primer mix. The amplified library underwent three rounds of purification using the Agencourt^®^ AMPure^®^ XP reagent. The library was then quantified using the Bioanalyzer High Sensitivity DNA chip (Agilent Technologies Inc, Santa Clara, CA, United States) followed by normalization to 12 to 25 pM for template preparation on the Ion One Touch (Life Technologies, Guilford, CT, United States).

The clonal amplification of the DNA libraries on the Ion Sphere Particles (ISPs) was carried out using emulsion PCR and the subsequent isolation of templated ISPs was performed using Ion OneTouch ES (Life Technologies, Guilford, CT, United States). Samples with polyclonal percentage of less than 35% and enriched, template–positive ISPs of more than >80% were subjected to sequencing on the Ion Torrent Personal Genome Machine (PGM^TM^) in a 318^TM^ chip (one sample per chip) using Ion Torrent PGM Sequencing 200 kit V2 (Life Technologies, Guilford, CT, United States).

### Validation Using Sanger Sequencing

Somatic variants identified were validated using Sanger sequencing method. Primers were designed using PrimerQuest Tool by Integrated DNA Technology (IDT, United States). The lists of the primers’ sequences used for validation were compiled in Supplementary Table 1. Briefly, PCR products were generated and cycle sequencing was performed using the Big Dye Terminator V3.1 reagent (Life Technologies, Guilford, CT, United States). The cycle sequencing products were then purified using ethanol precipitation and sequencing was carried out using the ABI 3500 Genetic Analyzer (Life Technologies, Guilford, CT, United States). The results were analyzed using the Basic Local Alignment System Tools (BLAST).

### Site-Directed Mutagenesis

The plasmid containing the full length cDNA human Adenomatous Polyposis Coli (hAPC) cloned in PCMV6 with MYC-DDK-tagged was purchased from OriGene (catalog number RC226492, Origene, Rockville, MD, United States). The mutations were incorporated into the hAPC and PCMV6 constructs by QuickChange Site-Directed Mutagenesis kit (Stratagene, Agilent Technologies, United States) and primers were designed to target specific site using the sequences as follows: APC p.R805^∗^; 5′-tctgacctattatcatcatgtcaattggtgtcaaaaacataatcac-3′ and 5′-gtgattatgtttttgacaccaattgacatgatgataataggtcaga-3′; APC p.Q1378^∗^; 5′-gtggggtctcctaaacatagtgttcaggtggactt-3′ and 5′-aagtccacctgaacactatgtttaggagaccccac-3′. We then screened for transformed colonies using Sanger sequencing at appropriate regions. Primer walking was performed for wild type (WT) construct in order to confirm that there were no other mutations present. Primer walking was performed by 1st Base Sdn. Bhd (Selangor, Malaysia).

### mRNA Expression Analysis

For cDNA syntesis, 1 μg of total RNA were reverse transcribed using high capacity RNA-to-cDNA Kit (Applied Biosystems, Foster City, CA, United States) according to the manufacturer’s protocol on the Veriti^TM^ Thermal Cycler (Applied Biosystems, Foster City, CA, United States). Target gene expression was determined via quantitative Real-Time PCR analysis using Taqman Fast Advanced Master Mix (Applied Biosystems, Foster City, CA, United States) on 7500 Fast Real-Time PCR machine (Applied Biosystems, Foster City, CA, United States). The expression values of the genes of interest (APC; Hs01568269) in each of the transfected cells of constructs plasmid were normalized to the expression value of the endogenous control, GAPDH (catalog no. 1391084). Relative fold change of the targeted gene levels between the transfection groups were determined by the 2^-ΔΔCT^. All experiment reactions were performed in triplicates.

### Western Blot and ELISA Analysis

Cells 293T were lysed in 1X RIPA buffer (Thermo Scientific, United States) and 10 μl protease inhibitor cocktail (Thermo Scientific, United States). After determination of protein concentrations by Bradford assay (Thermo Scientific, United States), equal amounts of cell lysates were separated by using mini-PROTEAN^®^ TGX^TM^ 4–20% 10W (Bio-Rad Laboratories Inc., United States). Separated proteins were transferred onto a 0.2 μm PVDF Trans-Blot^®^ Turbo^TM^ membrane (Bio-Rad Laboratories Inc., United States) for 30 min at 25 V using the Trans-Blot^®^ Turbo^TM^ Transfer System. The membrane was blocked with blocking buffer in Tris-buffered saline-Tween-20 (TBS-T) (Bio-Rad Laboratories Inc., United States) for 1 h at room temperature and incubated with mouse monoclonal antibody APC (A-3: sc-393704) (Santa Cruz Biotech, San Diego, CA, United States) with dilution 1:1000 at 4°C for overnight. The following day, the membranes were probed with secondary antibody goat anti-mouse IgG-HRP (sc-2005) diluted to 1:2000 and bands were visualized by enhanced chemiluminescence Clarity^TM^ and Clarity Max^TM^ Western ECL Blotting Substrates (Santa Cruz Biotech, San Diego, CA, United States). Imaging was performed using the ChemiDOC^TM^ MP Imaging System (Bio-Rad Laboratories Inc., United States).

Once primer walking confirmed there were no other mutations present in our APC WT plasmid, ELISA was performed to confirm the transfection by detecting anti-DDK signal. The protein lysate from transiently transfected cells were coated on PVC microtiter plate. ELISA was performed according to the manufacturer’s instructions using antibody anti-DDK (Clone OTI4C5: TA50011-100) (catalog number RC203465, Origene, Rockville, MD, United States) and the absorbance was measured at 490 nm after adding TMB substrate (Thermo Fisher Scientific, Waltham, MA, United States) and the stop solution of 2 M sulfuric acid.

### Cell Viability Assay

The SW480 cells (3 × 10ˆ4) were plated and cultured overnight in a 96-well plate. On the next day, the cells were transiently transfected using 0.75 μl Lipofectamine 2000 (Invitrogen, Carlsbad, CA, United States) with 0.2 μg APC WT and APC mutant constructs. The viability rate was determined by PrestoBlue^TM^ Cell Viability reagent (Invitrogen, Carlsbad, CA, United States) at 48 h post-transfection according to the manufacturer’s protocol. The fluorescence was measured using a microplate reader SkanIt RE for Varioskan Flash 2.4 (Thermo Fisher Scientific, Waltham, MA, United States) at excitation/emission wavelengths of 560/590 nm. The experiment was performed in triplicates in three independent experiments.

### Clonogenic Assay

For the clonogenic assay, SW480 cells were seeded at a density of 1.5 × 10ˆ5 cells per well in 24 well plate in triplicates. On the following day, cells were transfected with 1 μg of plasmid constructs using 2.5 μl Lipofectamine 2000 and incubated for 48 h. After 48 h of transfection with plasmid constructs, 5000 cells from the pooled triplicates of 24 wells were plated in a 6-well plate in triplicate with complete media. The plates were swirled to ensure an even distribution of the cell. The cells were grown in 37°C incubator with 5% CO_2_ for 14 days with media replacement every 3 days. At day 14, the media was discarded and cells were washed twice with PBS. The colonies were fixed with 10% acetic acid for 30 min, removed and stained with 0.5% crystal violet solution for 1 h. In order to remove the excess staining, the plate was washed three times with tap water. Images of the stained plates were captured, and the cell colonies containing more than 50 cells were counted.

### Bioinformatics Analysis

#### Read Mapping and Variants Calling

Data from the sequencing runs were automatically transferred to the Torrent Server hosting the Torrent Suite Software v4.0.3 to process the raw voltage semiconductor sequencing data into the DNA base calls. The Torrent Suite Software 4.0.3 utilizes the Torrent Browser that includes TMAP alignment and Torrent Variant Caller for alignment and variant detection using Somatic Low Stringency default setting. The reads were aligned against the hg19 reference sequence.

#### Variants Annotation

Annotation of variants was performed using the ANNOVAR (Wang et al., 2010). Gene based annotation was performed against RefSeq Gene, UCSC Known Gene and ENSEMBL Gene. The variants were further annotated against the conserved region (phastConsElements46way), segmental duplication region (genomicSuperDups), alternative allele frequency in all subjects in the NHLBI-ESP project with 6500 exomes (esp6500_all), alternative allele frequency data in 1000 Genomes Project (1000g2012apr_all), dbSNP version 138 (snp138), CLINVAR (clinvar_20140211) and COSMIC version 68 on WGS data (cosmic68wgs). Protein impact prediction was also performed on Annovar using SIFT, PolyPhen2 HDIV, PolyPhen2 HVAR, LRT, MutationTaster, MutationAssessor, FATHMM, GERP++, PhyloP and SiPhy (using command ljb23_all). The Drug-Gene Interaction Database (DGIdb), a web resource that consolidates information describing drug-gene interactions and gene druggability was used to identify potentially actionable variants (Wagner et al., 2016). Mutation Mapper and Oncoprinter tools were used to create the lollipop plot and oncoprint diagram, respectively (Cerami et al., 2012; Gao et al., 2013).

Single nucleotide variations and indels were defined based on the following three conditions: (1) the number of uniquely mapped reads at the position should be two or more, (2) the average base quality (phred Q score) for the position should be at least 20, (3) the read-allele frequency at the position should be at least 5%, and (4) the variant coverage should be at least 20 (Cai X. et al., 2014). For detection of the somatic alterations (SNVs and indels) in the cancer tissues, we used the following conditions: (1) non-synonymous SNVs or indels in the cancer tissues, (2) the wild-type allele count should be 10 or more in targeted sequence of normal tissue (Han et al., 2013), and (3) the SNV allele variant reads < 5 in the targeted sequence of normal tissue.

#### Driver Gene Alterations and Pathway Analysis

IntOGen (Gonzalez-Perez et al., 2013) was used to identify driver gene alterations among the somatic alterations. The IntOGen pipeline integrates the results of tumor genomes analyzed with different mutation-calling workflows and is scalable to hundreds of thousands of tumor genomes. At the time of analysis, it includes OncodriveFM (Gonzalez-Perez and Lopez-Bigas, 2012), a tool that detects genes that are significantly biased toward the accumulation of alterations with high functional impact (FM bias) without the need to estimate background mutation rate, and OncodriveCLUST (Tamborero et al., 2013), which picks up genes whose alterations tend to cluster in particular regions of the protein sequence with respect to synonymous alterations (CLUST bias) (Gonzalez-Perez et al., 2013). Ingenuity Pathway Analysis (Qiagen, Valencia, CA, United States) was used to identify the involvement of the altered genes in CRC pathway.

### Statistical Analysis

Significance was determined using two-tailed Fisher’s exact tests calculated by GraphPad QuickCalcs^1^ accessed on March 18th, 2017.

## Results

### Clinical Information of Samples

The demographic features of the studied subjects are presented in **Table 1**. The median age was 66 years for the Dukes’ B patients and 58 years for the Dukes’ C patients. More female patients presented with Duke’s C and majority of the tumors were located at the distal colon. Majority of the CRC cases included in this study were microsatellite stable.

**Table 1:** Clinicopathological characteristics of CRC patients.

Sample ID	Gender/Age	Histological subtype	Stage		MS1
			Dukes’	TNM staging	
BT1	M/57	Well differentiated, Adenocarcinoma	B2	T3 N0 Mx	MSS
BT2	M/58	Well differentiated with lymphovascular invasion, Adenocarcinoma	B	T3 N0 Mx	MSS
BT3	M/66	Well differentiated, Adenocarcinoma	B	NA	MSS
BT4	M/71	Moderately differentiated, Adenocarcinoma	B2	T3 N0 Mx	MSS
BT5	F/76	Moderately differentiated, Adenocarcinoma	B2	T3 N0 Mx	MSS
BT6	M/70	Well differentiated, Adenocarcinoma	B	T3 N0 Mx	MSS
BT7	F/66	Moderately differentiated, Infiltrating adenocarcinoma	B	T3 N0 Mx	MSS
BT8	F/56	Moderately differentiated, Adenocarcinoma	B	T3 N0 M0	MSI
BT9	M/68	Moderately differentiated, Adenocarcinoma	B	T2 N0 Mx R0	MSS
BT10	M/44	Moderately differentiated, Adenocarcinoma	B2	T3 N0 Mx	MSS
CT1	F/70	Moderately differentiated, Adenocarcinoma	C	T3 N1 M0	MSS
CT2	M/54	Moderately differentiated, Adenocarcinoma	C	NA	MSS
CT3	F/53	Moderately differentiated, Adenocarcinoma	C	NA	MSS
CT4	F/72	Moderately differentiated with lymph node metastasis, Adenocarcinoma	C	NA	MSS
CT5	F/52	Moderately differentiated, Adenocarcinoma	C2	NA	MSS
CT6	F/58	Moderately differentiated with lymph node metastasis, Adenocarcinoma	C	NA	MSS
CT7	F/64	Invasive, moderately differentiated, Adenocarcinoma	C	T3 N2b Mx R0	MSS
CT8	F/40	Moderately differentiated, Adenocarcinoma with perforation.	C1	T4 N1 Mx	MSI
CT9	F/73	Well differentiated, Adenocarcinoma	C	T3 N0 Mx	MSS

*M, male; F, female; TNM, tumor-node-metastasis; NA, not available; MSI, microsatellite instable; MSS, microsatellite stable*.

### Technical Performance of the Ion AmpliSeq^TM^ Comprehensive Cancer Panel v2

The Ion AmpliSeq^TM^ Comprehensive Cancer Panel contains 16,000 primer pairs that cover all the exons of 409 most common cancer-associated genes (Supplementary Table 2). The average sample loading obtained was 83.4% (range 61–93%). The total reads ranged from 3,728,210–11,430,479 reads with an average read length of 109 bp. The details on the loading percentage, number of reads and sequenced bases for each of the samples are summarized in Supplementary Table 3. On average, the target base coverage for each sample at 100× is more than >85% and average for uniformity 92.18% (Supplementary Table 4).

### Summary of Identified Variants

**FIGURE 1:**
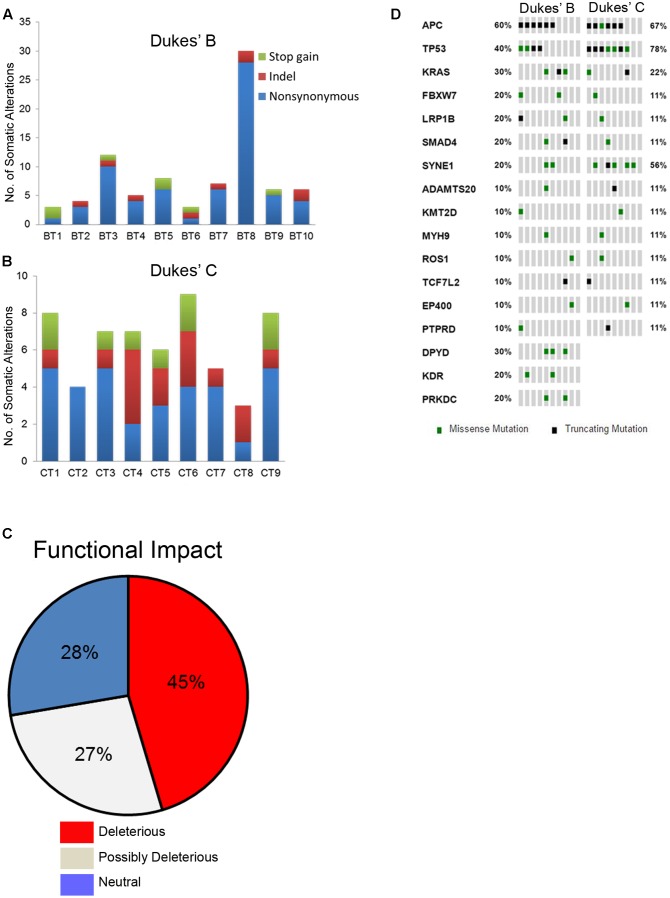
**(A,B)** Bar charts showing the number of somatic alterations identified in each patients according to respective groups. Indel in these bar charts including frameshift and non-frameshift substitution. **(C)** Pie chart showing the frequency of functional impact of gene mutations based on protein prediction score. **(D)** Oncoprint diagram illustrating the altered genes in at least two patients (Cerami et al., 2012; Gao et al., 2013).

**FIGURE 2:**
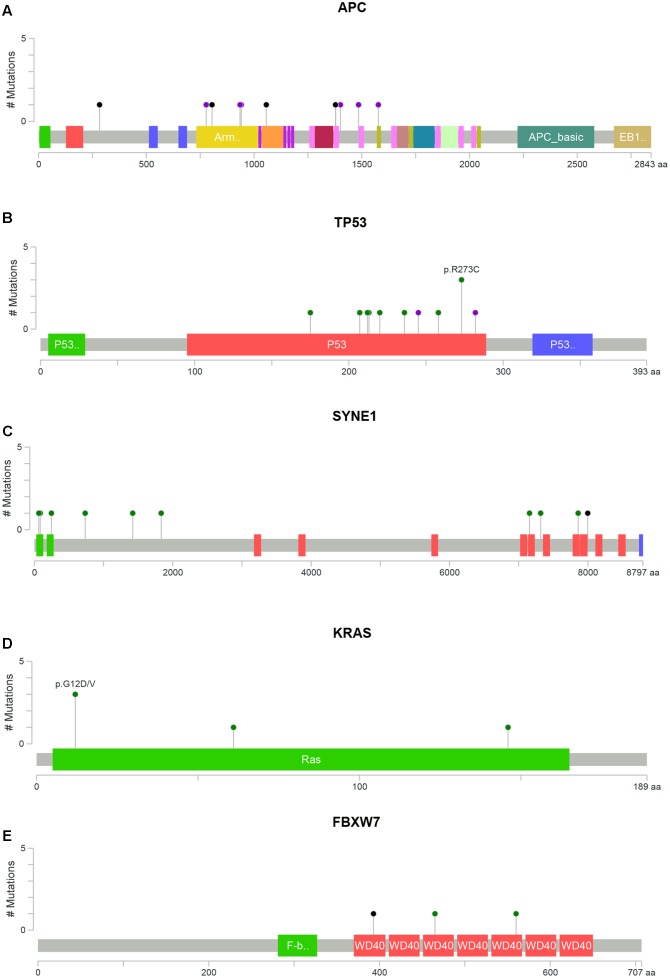
Distribution of somatic alterations on functional domains per gene. The alterations were represented by circle and colors; green (missenses), black (truncating alterations) and purple (others) while the length of the line represent the number of alterations identified at that codon. **(A)** Alterations detected in gene APC, **(B)** TP53, **(C)** SYNE1, **(D)** KRAS, and **(E)** FBXW7 (Cerami et al., 2012; Gao et al., 2013).

Overall, a total of 141 somatic non-synonymous variants were identified in 86 genes. All patients from both groups have at least three alterations among the 409 genes screened. In the Dukes’ B cases, we found 79 SNVs in 57 genes from all patients and five indels (an insertion and four deletions) in four genes from four patients (**Figure 1A** and Supplementary Table 5). Meanwhile, in the Dukes’ C cases, we identified 50 SNVs in 40 genes from all patients and seven indels (three insertions and four deletions) in five genes from five patients (**Figure 1B** and Supplementary Table 6). From the total of 141 variants, 64 (45%) were predicted to be deleterious, 38 (27%) were predicted to be possibly deleterious and 39 (28%) has neutral or low protein impact (**Figure 1C**). Notably, 17 genes, including the expected APC, TP53, KRAS, and FBXW7 genes were altered in at least two samples (**Figure 1D**). In both groups, the most commonly mutated genes were APC (12 patients), TP53 (11), SYNE1 (7 patients) and KRAS (5 patients). **Figure 2** illustrates the distribution of somatic alterations on functional domains of these genes.

*APC* alterations were the most common in our samples (12/19). Eight of the 13 *APC* alterations detected resulted in stop codons (p.R283^∗^, p.R805^∗^, p.E1057^∗^, p.Y935fs, two alterations of p.Q1378^∗^ and p.E1379^∗^ for each); four frameshift deletions (p.N778fs, p.I1401fs, p.A1485fs, and p.E1577fs) and one frameshift insertion (p.E941fs) (**Table 2**). We also identified *TP53* alterations in 11 out of 19 samples at known hotspot locations in exon 5 (p.R175H), exon 6 (p.F212V, p.R213^∗^, p.Y220C), exon 7 (p.Y236S, p.G245V, p.E258G) and exon 8 (three alterations of p.R273C, p.R282W). More *TP53* alterations were identified in Dukes’ C compared to Dukes’ B (Dukes’ C, *n* = 7 vs. Dukes’ B, *n* = 4), however, it did not reach statistical significant (*p* = 0.168). In addition, our results also showed 10 alterations in the SYNE1 gene in seven samples (nine missense alterations and one stop gain; p.L1833V, p.K1421T, p.P734L, p.R85L, p.R7157C, p.A7318E, p.L64F, p.A246T, p.G7860E, and p.W7998^∗^) (**Table 3**). On the date we performed the analysis, all of the SYNE1 alterations were not yet reported in snp138 or other CRC cases and predicted to be driver variants. *KRAS* alterations were identified in five out of 19 patients: three in Dukes’ B and two in Dukes’ C. These were all missense alterations located at the known hotspot region at exon 2 (p.G12D, 2 alterations of p.G12V), exon 3 (p.Q61H) and exon 4 (p.A146T). Three of the 19 samples (16%) had alterations in *FBXW7* found at known mutational hotspot in exon 8 (p.R393X), exon 9 (p.R465C) and exon 11 (p.F560N). In this study, two missense FBXW7 alterations were identified in two patients with Duke’s B and a nonsense alteration found in one patient with Dukes’ C CRC.

**Table 2:** Adenomatous polyposis coli (APC) somatic alterations in Dukes’ B and Dukes’ C CRC.

Sample ID	Exonic Function	snp138	Protein change	Exon	intOGen
BT4	Stopgain SNV	–	p.R283^∗^	9	Driver
BT8	Frameshift deletion	–	p.N778fs	16	Driver
CT4	Stopgain SNV	–	p.R805^∗^	16	Driver
CT1	Stopgain SNV	–	p.Y935fs	16	Driver
CT6	Frameshift insertion	–	p.E941fs	16	Driver
BT1	Stopgain SNV	–	p.E1057^∗^	16	Driver
BT5	Stopgain SNV	rs121913329	p.Q1378^∗^	16	Driver
CT9	Stopgain SNV	rs121913329	p.Q1378^∗^	16	
BT1	Stopgain SNV	rs121913326	p.E1379^∗^	16	Driver
BT9	Stopgain SNV	rs121913326	p.E1379^∗^	16	
CT3	Frameshift deletion	–	p.I1401fs	16	Driver
CT7	Frameshift deletion	–	p.A1485fs	16	Driver
BT7	Frameshift deletion	–	p.E1577fs	16	

*SNV, single nucleotide variation. Snp138, Single Nucleotide Polymorphism database (dbSNP) build 138*.

**Table 3:** *SYNE1* somatic alterations in Dukes’ B and Dukes’ C CRC.

Sample ID	Exonic function	snp138	Protein Change	Exon	intOGen
BT5	Stopgain SNV	–	p.W7998^∗^	132	Driver
BT5	Non-synonymous SNV	–	p.L1833V	41	Driver
BT8	Non-synonymous SNV	–	p.K1421T	33	Driver
BT8	Non-synonymous SNV	–	p.P734L	20	Driver
BT8	Non-synonymous SNV	–	p.R85L	5	Driver
CT2	Non-synonymous SNV	–	p.R7157C	116	Driver
CT5	Non-synonymous SNV	–	p.A7318E	119	Driver
CT6	Non-synonymous SNV	–	p.L64F	4	Driver
CT7	Non-synonymous SNV	–	p.A246T	9	Driver
CT9	Non-synonymous SNV	–	p.G7860E	129	Driver

*SNV, single nucleotide variation. Snp138, Single Nucleotide Polymorphism database (dbSNP) build 138*.

Among the 19 patients, 15 had alteration in *APC*, *KRAS*, and/or *TP53*; 10 had alterations in both *APC* and *TP53*, two patients had alterations in both *TP53* and *KRAS* or *APC*, *TP53*, and *FBXW7*. One patient harbored combined alterations in *KRAS* and *APC* or *KRAS* and *FBXW7* while one sample harbored a combination of *APC*, *TP53*, and *KRAS* alterations.

### Identification of Driver Gene Alterations

**FIGURE 3:**
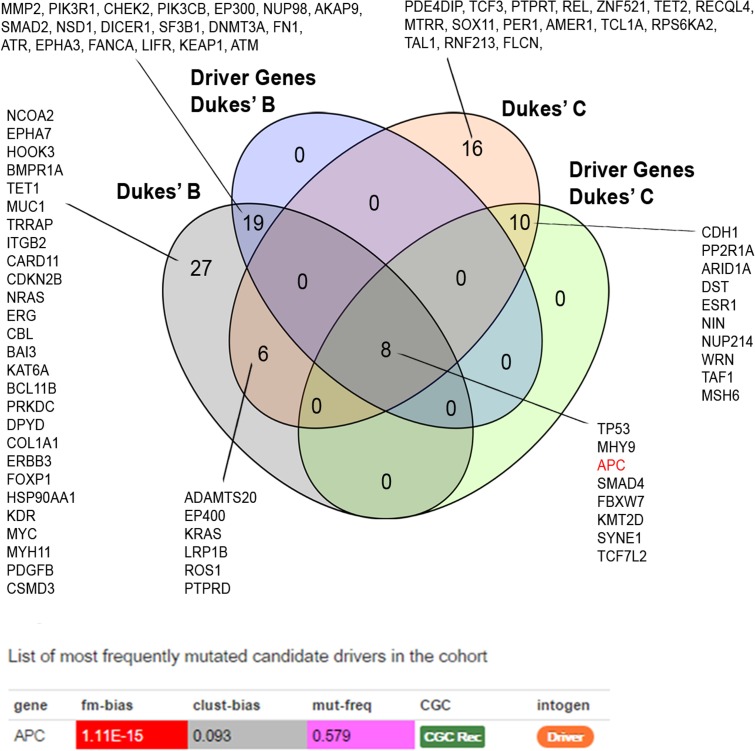
Venn diagram illustrating the overlapped altered genes. APC alterations were identified as the most frequent significant driver gene in our patients. Driver prediction was performed using IntOGen (Gonzalez-Perez et al., 2013).

We identified 37 out of 86 (43%) genes predicted as driver alterations and this included a total of 64 alterations. From this 64 alterations, 37 (58%) were identified in Dukes’ B whereby eight out of the 10 Dukes’ B patients have at least one candidate driver alteration. Meanwhile in Duke’s C, 27 (42%) alterations were identified in Dukes’ C where all Dukes’ C patients have at least one candidate driver gene alteration. Only the APC was identified as the significant driver gene in our patients. **Figure 3** shows the list of candidate driver genes in both Dukes’ stages.

### Druggable Somatic Variants

**FIGURE 4:**
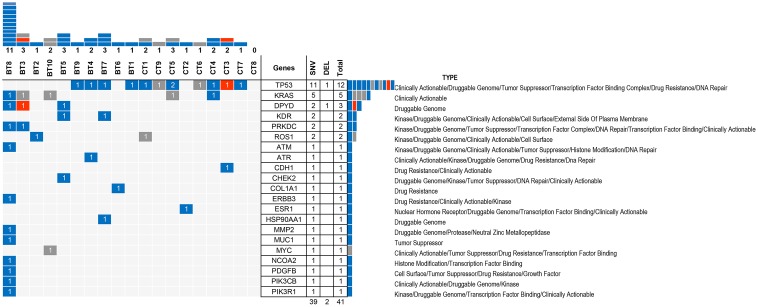
Druggable alteration patterns for 21 genes in 19 samples. The blue bars represent SNVs whereas the orange bars represent indels. The numbers inside the bars represents the number of alterations and the upper columns represent the number of alterations in each sample. BT8 exhibited the highest number of druggable alterations (11) while CT8 does not have any druggable target among the 409 genes. The right columns showed the number of alterations in each gene; TP53 has the highest number of druggable alterations (12), followed by *KRAS*, *DPYD*, *KDR*, *PRKDC*, and *ROS1*, whereas 15 other genes only exhibited one druggable alteration. SNV, single nucleotide variation.

Notably, almost all (10/10 and 8/9) of CRC patients in both groups harbored at least one actionable alteration that has been linked to a clinical treatment option or is currently being investigated in clinical trials for novel targeted therapies (**Figure 4**).

### Major Signaling Pathways Altered in CRCs

**FIGURE 5:**
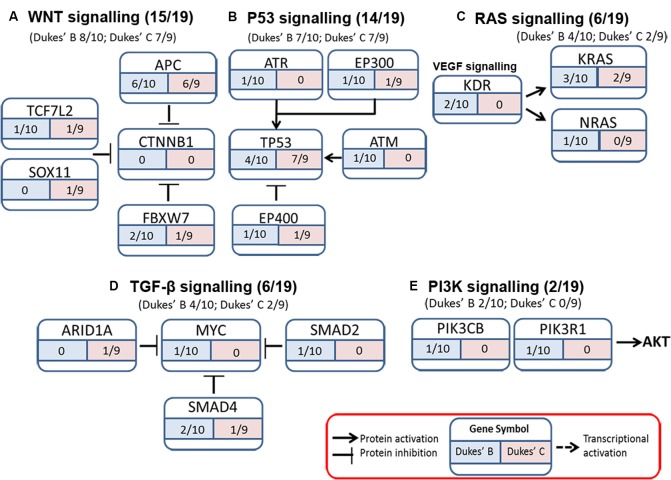
Somatic alterations found in this study were involved in five major signaling pathways known to be associated with CRC **(A)** WNT signaling, **(B)** P53 signaling, **(C)** RAS signaling, **(D)** TGF-β signaling, and **(E)** PI3K signaling.

**FIGURE 6:**
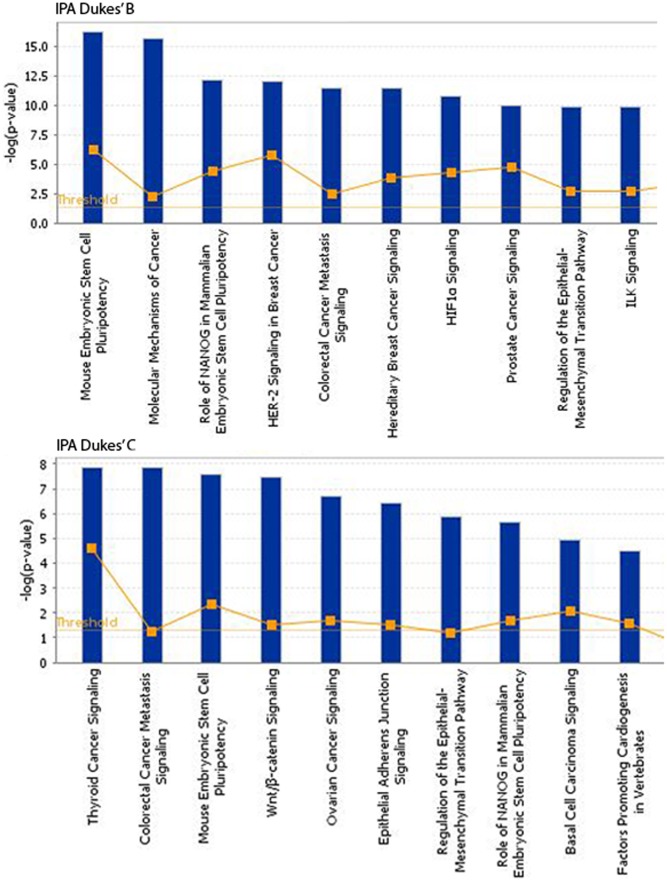
Top 10 significant canonical signaling pathways identified through IPA for both Dukes’ B and C. Data were analyzed through the use of QIAGEN’s Ingenuity^®^ Pathway Analysis (IPA^®^, QIAGEN Redwood City, www.qiagen.com/ingenuity).

**FIGURE 7:**
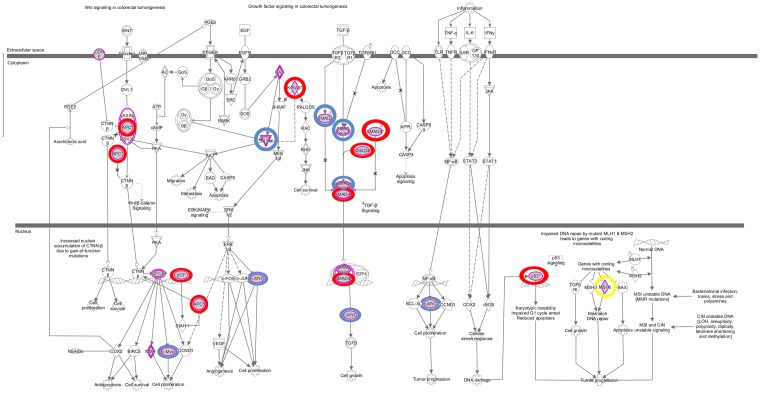
Colorectal cancer metastasis signaling pathway. This pathway ranked fifth and second significantly affected canonical pathway in Dukes’ B and C, respectively. The key altered genes identified in both groups were circled in red, genes only altered in Dukes’ B were circled in blue and gene only altered in Dukes’ C was circled in yellow. Data were analyzed through the use of QIAGEN’s Ingenuity^®^ Pathway Analysis (IPA^®^, QIAGEN Redwood City, www.qiagen. com/ingenuity).

The somatic alterations identified were then used to investigate the effect on the major signaling pathways in CRC (i.e., Wnt, P53, TGF-β, Ras, and VEGF signaling), by comparing the frequencies with which the genes involved in these pathways were altered (**Figure 5**). Our analysis revealed that Wnt signaling pathway (15/19) was the major pathway affected followed by P53 signaling (14/19), RAS signaling (6/19), TGF-β signaling (6/19), and PI3K signaling (2/19) (**Figure 5**). In addition, CRC metastasis signaling pathway was identified as the second most commonly altered canonical pathway in Dukes’ C, but ranked fifth in Dukes’ B (**Figure 6**). The involvement of altered genes in the CRC metastasis signaling pathway is illustrated in **Figure 7**.

### Validation by Sanger Sequencing

**FIGURE 8:**
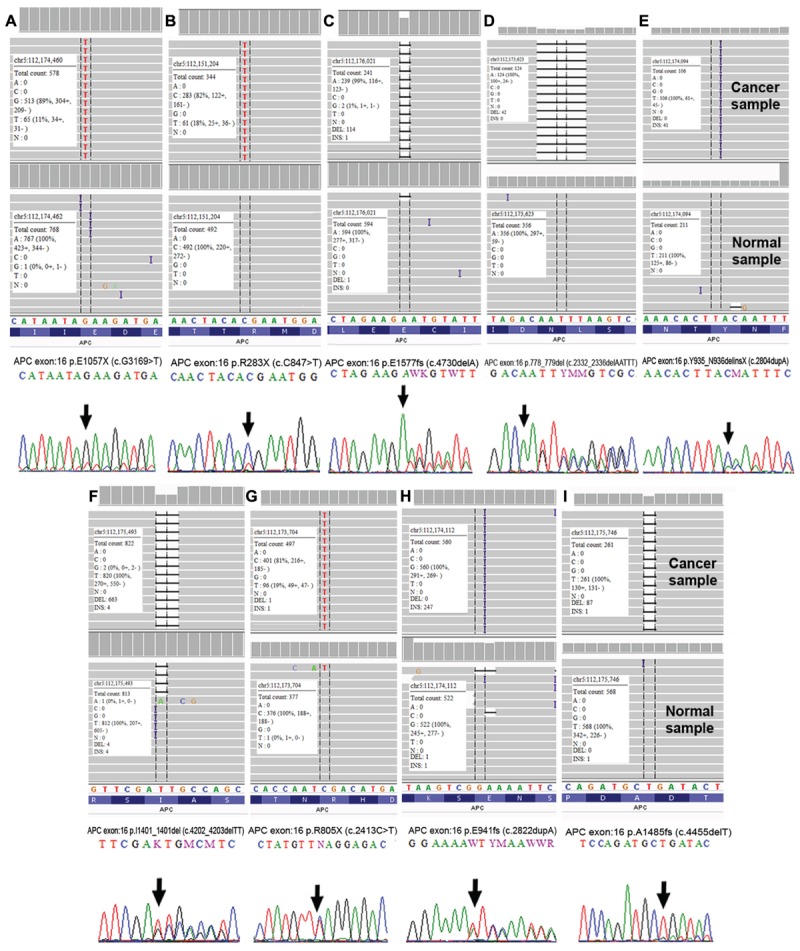
Validation of APC somatic alterations as visualized by Integrative Genomic Viewer (IGV) (Thorvaldsdóttir et al., 2013) and Sanger sequencing. **(A)** p.E1057X (c.G1369>T); **(B)** p.R283X (c.C847>T); **(C)** p.E1577fs (c.4730delA); **(D)** p.778_779del (c.2332_2336delAATTT); **(E)** p.Y935_N936delinsX (c.2804dupA); **(F)** p.I1401_1401del (c.4202_4203delTT); **(G)** p.R805X (c.2413C>T); **(H)** p.E941fs (c.2822dupA) and **(I)** p.A1485fs (c.4455delT).

We performed Sanger sequencing to validate selected somatic alterations in genes that have been previously reported in the CRC KEGG Pathway (Kanehisa et al., 2016), CRC TCGA data (Cancer Genome Atlas Network, 2012), mismatch repair genes and the most frequently altered genes in each groups. In total, 52 alterations from 23 genes were selected for validation and all were confirmed as true somatic mutations by Sanger sequencing. **Figure 8** shows the representative validated variants in APC gene.

### Functional Study of APC p.R805^∗^ and p.E1378^∗^

**FIGURE 9:**
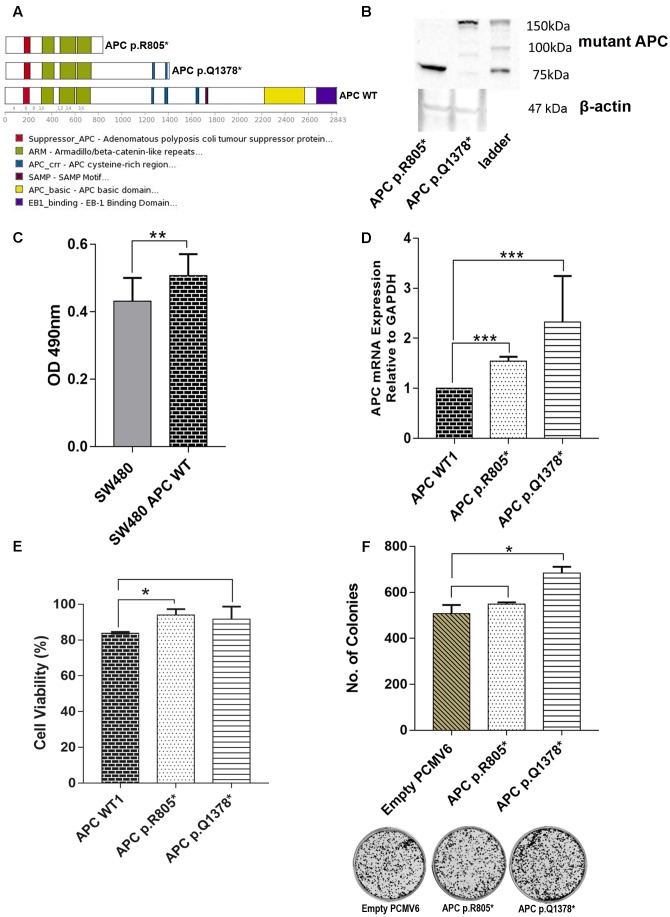
**(A)** Schematic representation of the full-length APC protein (2842 amino acids) and the truncated *APC* p.R805^∗^ lacking all domains involved in regulation of WNT/β-catenin signaling. *APC* p.Q1378^∗^ covered two of three 15-residue repeat domain important for binding to β-catenin. **(B)** Western Blot was performed to confirm the truncated protein’s size of *APC* p.R805^∗^ and *APC* p.Q1378^∗^ (around 89 and 151 kDa, respectively). **(C)** ELISA shows that there is significant difference between untransfected SW480 cell with transfected WT *APC* construct. **(D)** Expression levels of *APC* p.R805^∗^ and *APC* p.Q1378^∗^ in SW480 cell lines were compared to *APC* WT. The *APC* construct mutants are significantly up-regulated in SW480 cells. **(E)** Transfection of the *APC* p.R805^∗^ significantly promoted cell viability of SW480 cell line compared to the cells with *APC* WT but not in *APC* p.Q1378^∗^. **(F)** APC mutants promoted formation of colonies but only mutants APC p.Q1378^∗^ exhibits significant different when compared to empty vector and representative images of crystal violet stained colonies in each constructs were shown. Statistical analysis was performed using Student’s *t*-test. Error bars represent standard deviation (SD) (^∗^*p* < 0.05; ^∗∗^*p* < 0.01; ^∗∗∗^*p* < 0.001 and protein^∗^ = truncated protein).

We carried out functional analyses of selected *APC* variants to validate their involvement in CRC progression. Two truncated variants *APC* p.R805^∗^ which were found in CT4 patient and recurrent *APC* p. R1378^∗^ identified in two patients (BT5 and CT9) were selected. The schematic diagram of their effects on protein is shown in **Figure 9A**. We successfully created both of the mutant APC gene constructs and the screening of positive colonies at the appropriate region by Sanger sequencing (Supplementary Figures 1, 2). Western Blot confirmed the predicted effect that the variants resulted in truncated proteins (**Figure 9B**). Primer walking was confirmed that there were no other mutations identified in WT *APC* plasmid (Supplementary Figure 3). To prove that the WT *APC* was successfully transfected into SW480 cells, ELISA was performed to quantitate the amounts of anti-DDK receptors that could be detected on the cell surface. The result showed that there is a significant difference between untransfected SW480 cells with transfected SW480 with WT *APC* construct (**Figure 9C**). We also carried out mRNA expression analysis to assess the expression of the mutant RNAs. The mRNA expression levels of *APC* p.R805^∗^ and *APC* p.1378^∗^ were significantly up-regulated in SW480 cell lines compared to APC WT (**Figure 9D**). To determine whether mutant *APC* has growth-suppressive activity against SW480 cells, cell viability assay was conducted. Transfection of the *APC* p.R805^∗^ significantly promoted cell viability of SW480 cell lines compared to the cells with *APC* WT but not in *APC* p.Q1378^∗^ (**Figure 9E**). Meanwhile, *APC* WT significantly inhibits cell viability of SW480 cell line compared to empty vector (Supplementary Figure 4). *APC* p.R805^∗^ and *APC* p.1378^∗^ also promoted formation of colonies but only APC p.Q1378^∗^ exhibits significant different when compared to the empty vector (**Figure 9F**).

## Discussion

In this study, we screened for somatic alterations in 409 genes in CRC patients with Duke’s B and Duke’s C stage using the Ion Ampliseq Comprehensive Cancer Panel on the Ion Torrent PGM. We identified several cancer driver genes and illustrate their involvement in the CRC-related pathway. Overall, 63% of CRC patient’s harbored somatic alterations in the APC gene which included 13 alterations altogether and the majority were located in exon 16. To the best of our knowledge, at the time of analysis, four from the 13 alterations have not been reported in dbSNP version 138, in CRC or other cancers.

The APC gene is located on the long arm of chromosome 5 (5q22.2), consists of 16 exons (open reading frame from exon 2 to exon 16) and encodes a large protein ∼310 kD made of 2843 amino acids (Miki et al., 1992; Pineda et al., 2010). It is an important tumor suppressor known to play important roles in both hereditary and sporadic CRCs. Germline *APC* alterations are the main cause of familial adenomatous polyposis (FAP) which confers 100% lifetime-risk of CRC if left untreated (Groden et al., 1991). On the other hand, somatic alterations in this gene are detected in ∼70% of sporadic CRCs (Fearnhead et al., 2001; Narayan and Roy, 2003; Jaiswal and Narayan, 2008; Fearon, 2011) which is in concordance with our findings. *APC* alterations almost always result in a truncated protein product with abnormal function (Fearnhead et al., 2001). The truncations of the APC protein lead to the loss of β-catenin and/or axin binding sites and prevent β-catenin degradation, resulting in abnormally high levels of cytoplasmic and nuclear β-catenin in colon tumor cells (Munemitsu et al., 1995; Faux et al., 2004). In the nucleus, β-catenin interacts with members of the T-cell factor (Tcf)/lymphoid enhancer factor (LEF) family of transcription factors to activate transcription of Wnt target genes, including cyclin D1 and myc (Korinek et al., 1997) which promote proliferation and are associated with cellular transformation. Truncating *APC* alterations were detected in 68–95% of CRCs (Sieber et al., 2000; Christie et al., 2013). In our cases, truncating *APC* alterations were detected in eight out of 12 (66.7%) CRC patients with *APC* mutants (APC-mt). Currently, various treatments for *APC* mutant patients are being explored using multiple therapeutic approaches targeting both the Wnt-dependent and Wnt-independent functions of APC including reintroduction of APC, targeting the Wnt pathway downstream of the destruction complex and inhibiting targeted pathways regulated by APC. However, identifying *APC* alterations is pivotal for effective treatments, as loss of *APC* may cause resistance to certain drugs like cisplatin through enhanced DNA repair (Lesko et al., 2014).

Our study revealed APC as a significant driver gene in our patients and since the functional roles of the majority of APC variants have not been well investigated, we further performed functional analyses on two selected variants in this gene. Both were predicted by *in silico* algorithms as protein truncating variants which produce protein at different sizes and contain distinct domains which were *APC* p.R805^∗^ and *APC* p.Q1738^∗^. Western Blot analysis further confirmed the *in silico* prediction that these variants indeed produced truncated APC proteins. From the *in vitro* functional analysis, insertion of mutant *APC* p.R805^∗^ led to significant increase of the viability of cancer cells. Truncating *APC* p.R805^∗^ lacks all domains involved in regulation of WNT/β-catenin signaling. When this happens, it will activate the other Tcf members like cyclin D1 and myc, therefore causing uncontrolled of cell proliferation (Aoki and Taketo, 2007), as evident in our study. Meanwhile, the variant *APC* p.Q1738^∗^ showed a similar pattern although not significant. This might be because of the truncated site of this variant which has two out of three 15-residue repeat domains that still cover the important function for binding to β-catenin (Wang et al., 2014). In addition, based on the clonogenic assay, we also demonstrated the ability of both variants in driving *in vitro* cell transformation. The pathogenicity of *APC* variants on different protein domain might have different effect and is worth investigating for future studies.

We also identified alterations in other known CRC genes such as TP53, KRAS, FBXW7, and SMAD4, as reported by others (Cancer Genome Atlas Network, 2012; Han et al., 2013; Yu et al., 2014; Bai et al., 2015). TP53 gene, which is located at the long q arm of chromosome 17 at position 13, is altered in about 60–70% of CRCs and is important in cell cycle arrest and apoptosis processes (Fearon, 2011). Consistent with our findings, most *TP53* alterations were clustered in the TP53 DNA binding domain, which encompasses exon 5 through 8 and also a critical region for the transcription factor functions of the gene (Pfeifer and Besaratinia, 2009; Cai Z.X. et al., 2014). Majority of *TP53* alterations identified in our study were missense alterations and this is supported by other CRC studies (Cai Z.X. et al., 2014; Dallol et al., 2016). Most *TP53* missense alterations lead to the substitution of a single amino acid in the P53 protein that can be stably expressed in cancer cells (Muller and Vousden, 2014). These alterations generally lead to a loss or diminution of the wild type activity of TP53 and mutant proteins become inactive and lose the ability to transactivate downstream target genes that regulate cell cycle and apoptosis (Muller and Vousden, 2014).

Inactivation of TP53 gene is one of the most common events in CRC and plays a vital role in the tumourigenesis of colorectal epithelial cells. TP53 alterations are identified as late events in CRC development, with a loss of TP53-mediated apoptotic pathways as an important factor in the progression from an adenoma to a malignant tumor (Smith et al., 2002). The frequency and spectrum of *TP53* alterations were associated with the different grades, stages and locations of the tumor (Mahdavinia et al., 2008). A study by Mahdavinia et al. (2008) found that the frequency of *TP53* mutations significantly increased with tumor stages (36/94, 38.3%; 33/64, 51.5%, and 15/23, 65.2% in Stage B, C, and D, respectively) and this is also reflected in our finding. Further corroborating our finding, *TP53* alteration also exhibit location-based pattern, whereby its frequency is higher in distal as compared to proximal tumors (Samowitz et al., 2002; Russo et al., 2005) (44.7 vs. 55.3% and 35% vs. 45%, respectively). Researchers are still debating the role of *TP53* alterations as prognostic indicators, as some have reported that overexpression of *TP53* with elevated serum carcinoembryonic antigen (CEA) or carbohydrate antigen 199 (CA199) levels is significantly associated with poor outcome [median time from progression to death (TTD) for CEA ≥ 5 U/ml and P53 positive was 23.2 months (95% CI 17.1–29.3), and that of CA199 ≥ 27 U/ml and P53 positive was 15.2 months (95% CI 1.9–28.5)] (Cui et al., 2016), whereas others have found *TP53* mutations to have little or no prognostic value at all (Soong et al., 2000; Conlin et al., 2005).

SYNE1 gene is one of the biggest genes in the human genome, containing 147 exons that encode a 27,652 kb messenger RNA and 8,797 amino-acid-long protein (Gros-Louis et al., 2007). It is located at the long arm of chromosome 6 at position 25. This gene encodes a spectrin repeat containing the nuclear envelope 1 protein expressed in skeletal and smooth muscle and peripheral blood lymphocytes that localizes to the nuclear membrane (Lee et al., 2013). SYNE1 alterations are linked to cerebellar ataxia and have been associated with lung, ovarian, and CRCs (Doherty et al., 2010). With regards to epigenetics, hypermethylation of SYNE1 has been identified as a key factor in colitis-associated cancer (CAC) carcinogenesis and is a potential biomarker to identify patients at higher risk of CRC (Papadia et al., 2014). Long-standing ulcerative colitis (UC) and Crohn’s colitis are linked to an increased risk of developing CRC (Ekbom et al., 1990; Papadia et al., 2014). Melotte et al. (2015) reported methylated SYNE gene as one of the promising markers for CRC detection. SYNE1 is one of the frequently altered genes in our CRC patients (7/19) and this is concordant with a study by Yu et al. (2014) which reported 28/160 (17.5%) cases with alterations. SYNE1 also have been reported in glioblastoma (GBM) where these alterations were significantly correlated with the overexpression of several known GBM survival genes (Masica and Karchin, 2011) and the polymorphism of *SYNE1* (rs2295190, G-to-T change) was associated with an increased risk of invasive ovarian cancer, with a per-T-allele odds ratio (OR) of 1.24 [95% confidence interval (CI), 1.06–1.44, *P* = 0.006] (Doherty et al., 2010).

The TCGA project showed that the *KRAS* and *FBXW7* alterations rates in CRCs were 42 and 17%, respectively (Cancer Genome Atlas Network, 2012). KRAS gene is located on the short arm of chromosome 12 and encodes a 21-kD protein (Smith et al., 2002). Alterations in this gene lead to increased and unregulated cellular proliferation and malignant transformation (Smith et al., 2002). Even though our sample set had a lower frequency of *KRAS* alterations than expected (26%), we found that this gene had the commonest oncogenic codon 12 alterations affecting glycine 12 residue (two alterations of p.G12V; one alteration of p.G12D) as similarly reported with by other studies (Cancer Genome Atlas Network, 2012; Cai Z.X. et al., 2014; Dallol et al., 2016). KRAS p.G12V was reported as a biomarker for poor prognosis in resected non-small cell lung cancer (NSCLC) as it exhibited a worse overall survival (OS) and higher recurrence rates (Renaud et al., 2015). In CRC, KRAS p.G12V was identified in both primary and metastasis samples but the difference was not significant (Neumann et al., 2009). However, a recent clinical study found that the KRAS p.G12V alteration enhances metastases to lymph nodes, an indication of its higher aggressiveness in CRC animal model (Alamo et al., 2015). With regards to precision medicine, this alteration is reported to confer reduced sensitivity against cetuximab or panitumumab (anti-EGFR antibodies) (Di Fiore et al., 2007; Peeters et al., 2013), which is the main treatment for metastatic CRC. Hence, both agents should only be introduced into patients with RAS wild-type CRC. Alterations in other positions, such as codon 61 (p.Q61H) and 146 (p.A146T) have also been reported. However, these alterations account for a minor proportion of one to four percent of KRAS alterations, and their clinical relevance in CRC still remains unclear (Neumann et al., 2009; Tong et al., 2014).

FBXW7 is a potential tumor suppressor that regulates ubiquitination and proteolysis of multiple targets (Cai Z.X. et al., 2014). The gene is located on chromosome 4 on the short arm at position 31 and encodes for a subunit of an ubiquitin protein ligase that regulates the levels of many important oncoproteins such as Cyclin E, Notch 1, c-Myc and other proteins (Wang et al., 2012; Zheng et al., 2016). FBXW7 protein domain structure consists of seven WD4 repeats, which can form eight-bladed, barrel-shaped β-propeller-like binding pockets for its substrates (Orlicky et al., 2003; Hao et al., 2007). FBXW7 alterations impair Cyclin E degradation and are associated with decreased genetic stability and impaired growth regulation, contributing to the progression of CRC (Akhoondi et al., 2007). Low FBXW7 expressions in tumor as compared to normal tissues were significantly correlated with poor prognosis of overall survival in CRCs and esophageal cancer (EC) patients (Iwatsuki et al., 2010; Kurashige et al., 2012). A similar pattern of low overall survival was also found in early stage CRC patients with FBXW7 mutants but the results was not significant when compare to wild type patients (Chang et al., 2015). About 10 to 11% of FBWX7 alterations were found in most of CRCs studies (Eliana et al., 2013; Grim, 2014; Cai Z.X. et al., 2014; Bai et al., 2015). We found this alteration in 16% (3/19) of our non-metastatic CRCs patients.

We also observed several co-occurrence of alterations among the genes including APC, TP53, KRAS, and FBXW7. The most common combination of alterations in both APC and TP53 in our patients are in concordance with a previous study which involved European CRC patients (Smith et al., 2002) but contradicted with a report in Chinese CRCs (Cai Z.X. et al., 2014). Cai Z.X. et al. (2014) identified only four out of 93 CRC patients (4.3%) harbored *APC* and *TP53* alterations. In addition, alterations in *TP53* with *KRAS* were rare in European CRCs (17.5%) (Smith et al., 2002), which is also in concordance with our finding. Combination of *APC* and *KRAS* was also rare which was identified in only one of our patients and this is in concordance with study by Conlin et al. (2005). Bai et al. (2015) found the combination of *FBXW7* alteration with *KRAS* in five out of 91 CRC patients (5.5%) which were similar with our finding. However, their result on combination of *FBXW7* alteration with *APC* and *TP53* was contradicted with ours. We noticed that the co-occurrence of *APC*, *KRAS*, and *TP53* alterations in the same tumor was uncommon (5%) and this result concurs with those of other studies (Smith et al., 2002; Samowitz et al., 2007; Vasovcak et al., 2011). Collectively, these results indicate the wide variability in genetic alterations found in CRCs from different populations and highlights the need to further evaluate CRCs for common patterns of alterations.

Schell et al. (2016) recently revealed that in MSS tumors, the overall survival was roughly equivalent among *APC*, *APC*/*KRAS*, and *APC*/*TP53* altered groups, but lower in the *APC*/*KRAS*/*TP53* group. This seems to be reflected in one of our patients. Patient CT4, who harbored the triple alterations *APC*/*KRAS*/*TP53* and also a MSS tumor, had short overall survival of around 8 months. This patient was diagnosed with moderately differentiated adenocarcinoma of the colon with lymph node metastasis (Duke’s C) in June 2009, underwent sigmoid total colectomy in August 2009 and developed CRC metastasis to liver in September 2009. She died in February 2010.

Wnt signaling is the main perturbed pathway in the development of CRC carcinogenesis (Cancer Genome Atlas Network, 2012; Kandoth et al., 2013; Vogelstein et al., 2013; Ashktorab et al., 2015). This pathway was altered in 80% of CRC patients (Ashktorab et al., 2015), which is almost similar to our finding (78.9%). Among the genes altered in this pathway were APC (12/19), FBXW7 (3/19), TCF7L2 (2/19) as well as SOX11 (1/19) and these were similarly reported by Lee et al. (2014) in primary CRCs except for CTNNB1 and SOX9. We noticed that none of our samples have *CTNNB1* alteration as it is less common and have been reported in only six to nine percent of CRCs patients (Forbes et al., 2015). A major proportion of CRC patients (14/19; 74%) also harbored alterations in one or more genes in P53 signaling pathway including TP53 (11/19), ATM (1/19), and ATR (1/19) (encoding DNA damage proteins); EP300 (2/19) and EP400 (2/19) (encoding a P53 coactivator), which is consistent with findings from other studies (Lee et al., 2014; Yu et al., 2014).

In the Ras signaling pathway, aside from the dominant *KRAS* alterations (5/19), we had a patient with *NRAS* alteration (1/19) and this gene was previously reported to be altered in about five to eight percent of CRC patients (Forbes et al., 2015). Ras proteins are important in the signaling pathways that coordinate cell proliferation, differentiation, regulation of cell cycle as well as angiogenesis (Adjei, 2001; Zheng et al., 2016) and alterations in this gene are likely to result in constitutive activation and impaired regulatory functions (Downward, 2003).

SMAD4, SMAD2, MYC, and ARID1A were among the gene alterations found related to the TGF-β signaling pathway in our study. *SMAD4* and *SMAD2* alterations have been observed in 10–15% and 5% of CRCs, respectively (Fearon, 2011). *SMAD4* codes for a protein that is involved as a downstream regulator of the TGF-β signaling pathway (Bai et al., 2015), and acts as a trimer and forms complexes with the receptor-phosphorylated SMAD2 and SMAD3; where these heteromeric complexes enter the nucleus to regulate apoptosis and cell cycle (Woodford-Richens et al., 2001). *SMAD4* alterations caused the dysfunctional protein to interfere with proper signaling and gene transcription of target genes critical in cell cycle regulation (Bai et al., 2015). *ARID1A* alterations were reported in about nine percent in CRCs patient (Cancer Genome Atlas Network, 2012). In another study, inactivating mutations in *ARID1A* were shown to be frequent in microsatellite unstable CRC (Cajuso et al., 2014).

In the PI3K signaling pathway, we identified *PIK3R1* and *PIK3CB* alterations. Class I PI3Ks are the best-characterized enzymes and include a catalytic subunit (p110α, p110β, and p110δ) and a regulatory subunit (p85α, p55α, p50α, p85β, and p55γ). The regulatory subunit p85 α is encoded by *PIK3R1*, while the catalytic subunit p110β is encoded by *PIK3CB* (Yuan and Cantley, 2008). The PI3K pathway play crucial roles in regulating cellular processes including protein synthesis, cell growth, proliferation, angiogenesis, cell cycle and survival (Baselga, 2011). PIK3R1 alterations and p85α loss were associated with PI3K pathway activation and increased oncogenic potential (Cizkova et al., 2013), while PIK3CB gene is implicated in *PTEN*-deficient tumourigenesis (Wee et al., 2008).

Cancer is a heterogenous disease and for the application of precision medicine it is vital to have the exact genetic information of the individual tumor. A number of gene markers are already guiding treatment decisions in daily practice. For instance, the identification of *KRAS* mutation at codons 12 and 13 in metastatic CRC predicts the lack of benefit from EGFR-targeted antibodies (Van Cutsem et al., 2011), and for the non-small cell lung cancer patients with *EGFR* del746_A750 or L858R, they are very responsive to EGFR tyrosine kinase inhibitors (Mok et al., 2009). Most recently, the novel PARP inhibitor olaparib is indicated for treating advanced ovarian cancer patients who have mutations in *BRCA1* or *BRCA2* (Munroe and Kolesar, 2016) and also the identification of *KDR* alteration as a novel predictive biomarker of exceptional response to low dose regorafenib reported by Loaiza-Bonilla et al. (2016) in patients with advanced CRC.

## Conclusion

We have successfully profiled the gene alterations of a number of our local CRC patients and almost all patients harbored actionable mutations. Individualized cancer gene sequencing may be the next critical step in improving treatment options and increasing patient survival by selecting the appropriate therapeutic regime. By identifying gene alterations in individual cancers, specific treatments targeted against the detected gene alterations may prove to have greater benefits for cancer patients.

## Author Contributions

S-NA performed the experiments and data analysis. N-SM was involved in data interpretation, drafting the manuscript and overseeing the experiments. KS was heavily involved in data analysis. SS and MI were involved in optimization of library preparation and sequencing. NA was involved in functional analyses. NM and RJ were involved in critical review of the manuscript. IS and LM are colorectal surgeons involved in specimen collection and IR is a pathologist. All authors read and approved the final manuscript.

## Conflict of Interest Statement

The authors declare that the research was conducted in the absence of any commercial or financial relationships that could be construed as a potential conflict of interest.

## Footnotes

1 http://www.graphpad.com/quickcalcs/contingency2/
