# Supplementary material for: Molecular Characterization of Somatic Alterations in Dukes’ B and C Colorectal Cancers by Targeted Sequencing
Source: Front Pharmacol. 2017 Jul 18;8:465. doi: 10.3389/fphar.2017.00465 (PMC5513919; doi:10.3389/fphar.2017.00465)
Supplement: Supplementary file 2 [file Image_1.PDF]

Supplementary Material

Molecular Characterisation of Somatic Alterations in Dukes' B & C Colorectal Cancers by Targeted Sequencing

Shafina Nadiawati Abdul[1], Nurul-Syakima Ab Mutalib[1*], Khor Sheau Sean[2], Saiful Effendi Syafruddin[1], Muhiddin Ishak[1], Ismail Sagap[3], Luqman Mazlan[3], Isa Mohamed Rose[4], Nadiah Abu[1], Norfilza Mohd Mokhtar[5] and Rahman Jamal[1*]

*** Correspondence:** Corresponding Author: NSAM, syakima@ppukm.ukm.edu.my and RJ, rahmanj@ppukm.ukm.edu.my

Supplementary Figures

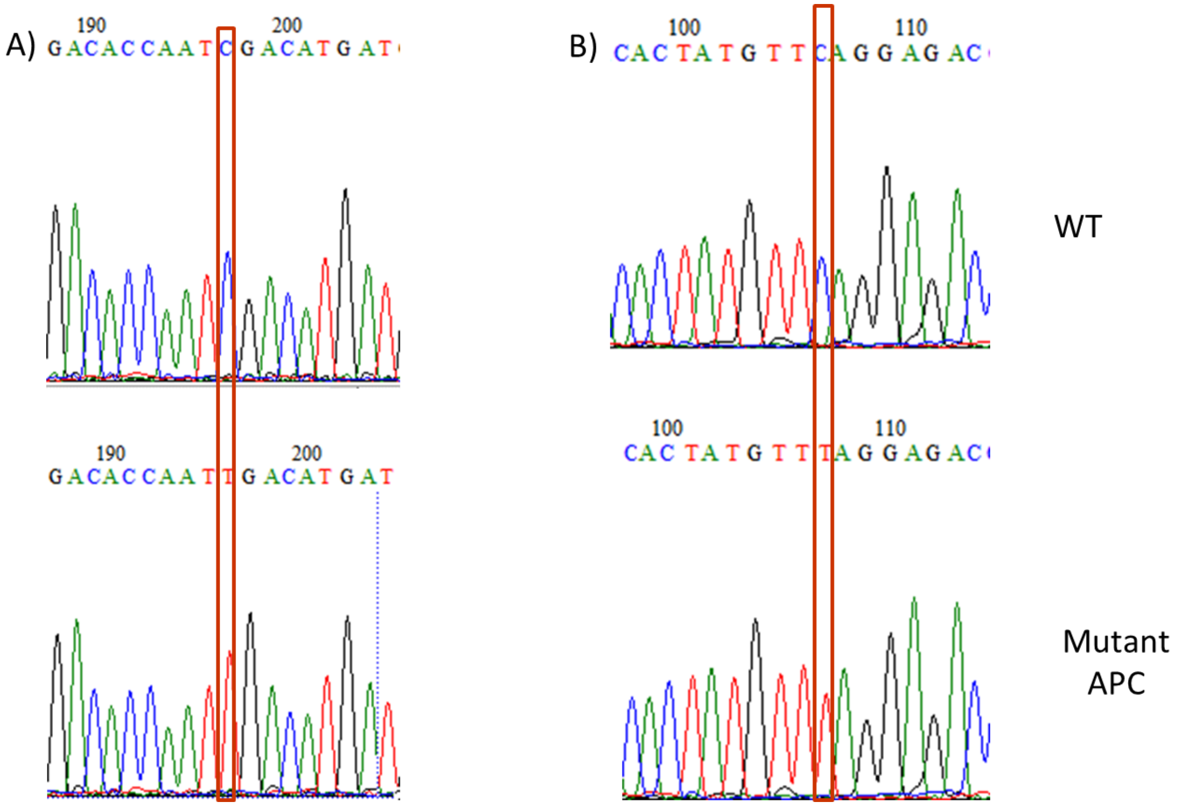

Supplementary Figure 1. Confirmation of site directed mutagenesis in A) APC p.R805* (c.2413C>T) and B) APC p.Q1378* (c.4132C>T) sequences compared with WT APC.

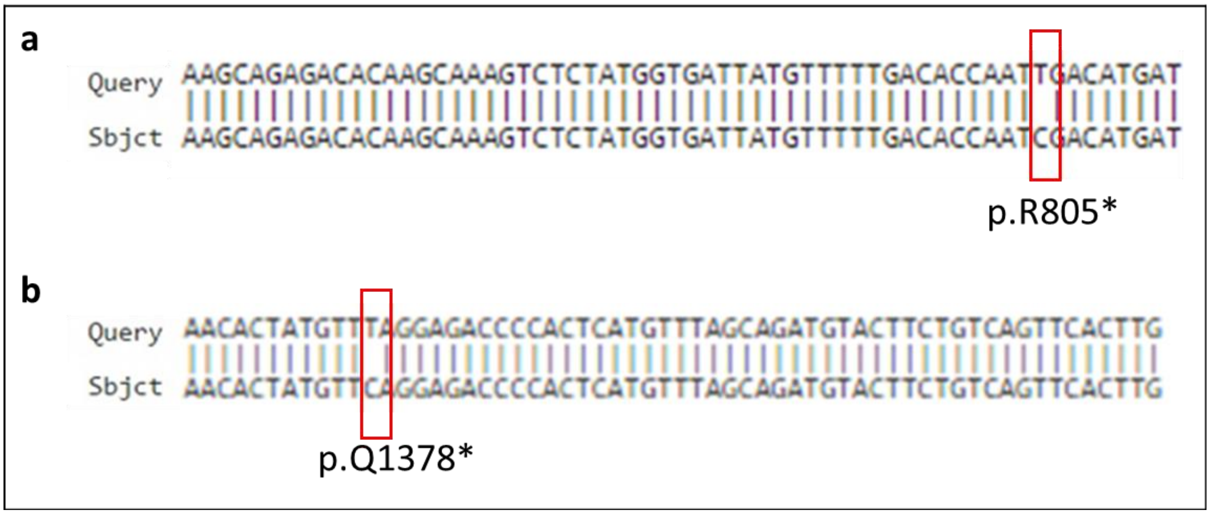

Supplementary Figure 2. Sequence alignment of (A) p.R805* and (B) p.Q1378* compared to the WT *APC*. All results showed that the mutations occur at correct place.

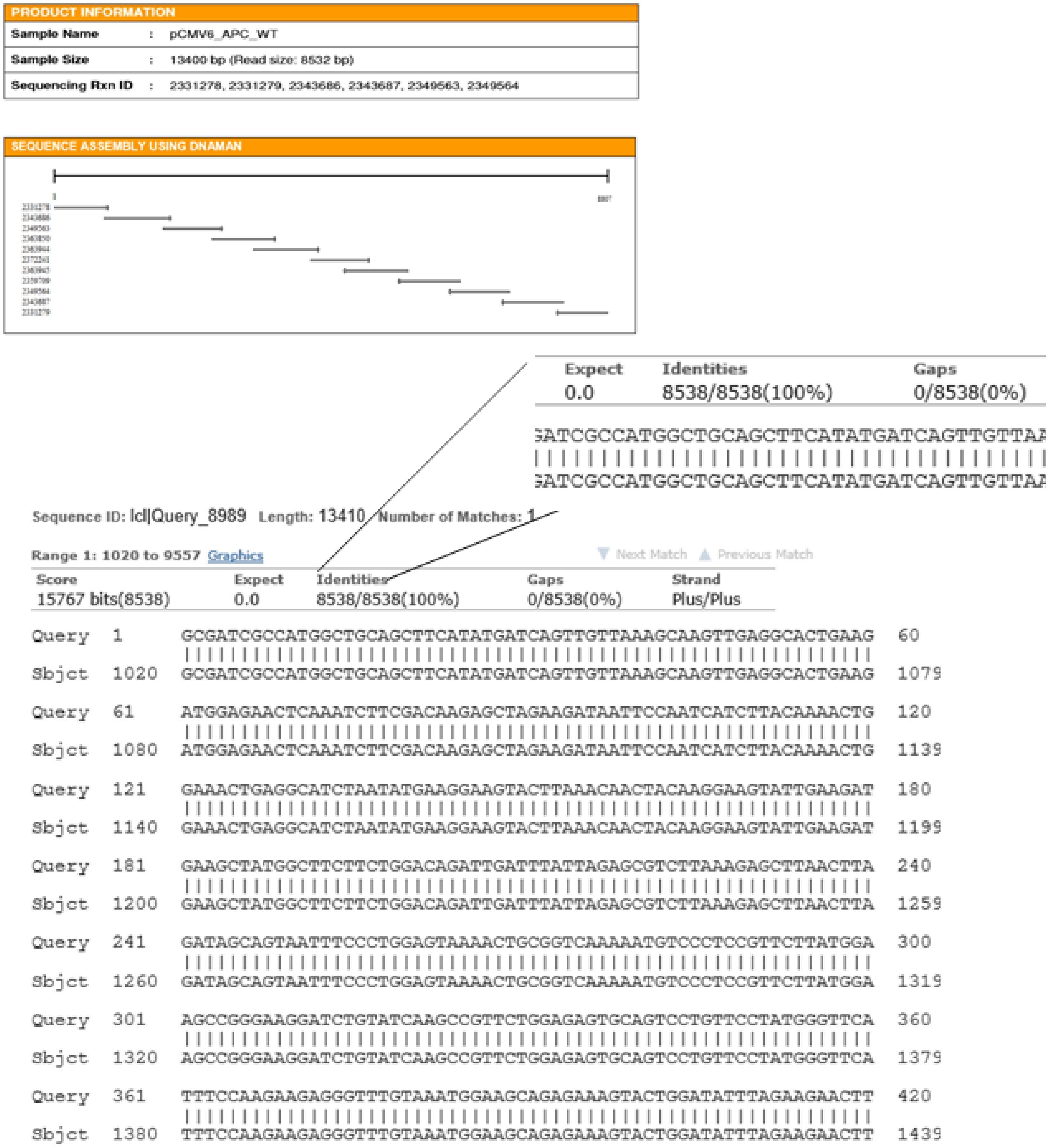

Supplementary Figure 3. Primer walking of full length APC WT construct. Total size insert APC gene is around 8.5 kb and 100% is identical.

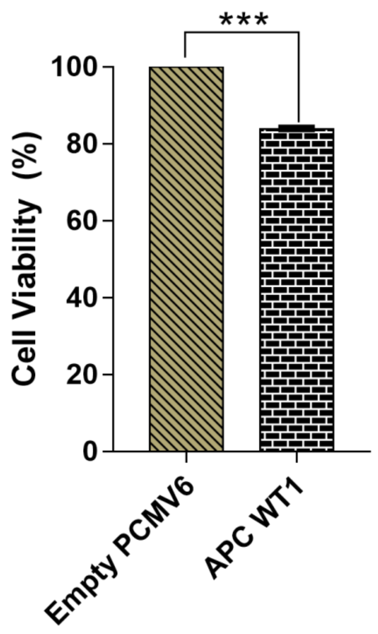

Supplementary Figure 4. Transfection of the *APC* WT significantly inhibits cell viability of SW480 cell line compared to empty vector. This confirmed the APC role as tumour suppressor gene.
